# A systematic review of dimensions evaluating patient experience in chronic illness

**DOI:** 10.1186/s12955-019-1084-2

**Published:** 2019-01-21

**Authors:** Bastien Forestier, Emmanuelle Anthoine, Ziad Reguiai, Cécile Fohrer, Myriam Blanchin

**Affiliations:** 1 UMR U1246 SPHERE “methodS in Patient centered outcomes & HEalth REsearch”, Université de Nantes, Université de Tours, INSERM, Nantes, France; 2 Pôle de santé publique, CHU de Nantes, Nantes, France; 3 Service de dermatologie, Polyclinique Courlancy, Reims, France; 4 Service d’hématologie clinique, CHU de Strasbourg, Strasbourg, France

**Keywords:** Chronic illness, Patient reported outcome measures, Patient reported experience measures, Patient-centered care, Patient experience, Systematic review

## Abstract

**Background:**

Living with a chronic disease often means experiencing chronic treatments and regular multidisciplinary monitoring as well as a profound life-changing experience which may impact all aspects of a patients life. The patient experience of chronic disease is frequently assessed by patient reported measures (PRMs) which incorporate patients perspectives to better understand how illness, treatment and care impact the entirety of a patient’s life. The purpose of this review was to collect and review different kinds of available PRM instruments validated for chronic patients, to produce an inventory of explored concepts in these questionnaires and to identify and classify all dimensions assessing chronic patients experience.

**Methods:**

A systematic review of PRM instruments validated for chronic patients was conducted from three databases (Medline, the Cochrane library, and Psycinfo). Articles were selected after a double reading and questionnaires were classified according to their targeted concept. Then, all dimensions of the questionnaires were clustered into different categories.

**Results:**

107 primary validation studies of PRM questionnaires were selected. Five kinds of instruments were recorded: 1) Questionnaires assessing health related quality of life or quality of life; 2) Instruments focusing on symptoms and functional status; 3) Instruments exploring patients’ feelings and attitude about illness; 4) Questionnaires related to patients’ experience of treatment or healthcare; 5) Instruments assessing patients attitudes about treatment or healthcare. Twelve categories of dimensions were obtained from these instruments*.*

**Conclusions:**

This review provided an overview of some of the dimensions used to explore chronic patient experience. A large PRM diversity exists and none of the reviewed and selected questionnaires covered all identified categories of dimensions of patient experience of chronic disease. Furthermore, the definition of explored concepts varies widely among researchers and complex concepts often lack a clear definition in the reviewed articles. Before attempting to measure chronic patient experience, researchers should construct appropriate instruments focusing on well-defined concepts and dimensions encompassing patient’s personal experience, attitude and adaptation to illness, treatment or healthcare.

**Electronic supplementary material:**

The online version of this article (10.1186/s12955-019-1084-2) contains supplementary material, which is available to authorized users.

## Background

Over the past two decades, greater recognition of patients’ point of view has been facilitated by patient reported measures (PRMs) which are directly reported by the patient without interpretation of their responses by a clinician or anyone else [1]. Patient reported experience measures (PREMs) are a class of PRMs used to capture patient experience of health care or health service [2]. If increased attention has been paid to the measurement of “patient experience” as a quality indicator of patient care and safety for accreditation [3, 4], there has also been a rapid expansion in the use of the term “patient experience” in the area of clinical practice and research [3]. The need for a patient-centered approach has been recognized for assessment of the impact of disease and treatment in a patients life. Patient reported outcome measures (PROMs) is a broad family of measures consisting of a direct report by the patient of health outcomes associated with illness or treatment. PROMs include assessment of symptoms, functional status, health-related quality of life (HRQOL), satisfaction and adherence to medication, and various psychological, physical, social aspects [4–6]. PREMs and PROMs are currently distinguished in the literature since the development and use of measures of patient reported experiences have often been carried out in isolation from work on patient reported outcomes [7]. In the field of chronic disease, patient experience often includes experience of chronic treatments, health practices and regular multidisciplinary monitoring as well as a profound life-changing experience which may impact all aspects of a patients life, inside and outside of care. The umbrella term “patient experience” for chronic diseases is somewhat confusing, and should therefore not be limited to patient healthcare experience as both PREMs and PROMs are of interest. Even if it appears that each class of PRMs overlap with the other, each one does not capture the whole complexity of chronic patient experience with chronic disease [7]. Therefore, chronic patient experience should simultaneously take into consideration PREMs, providing insight into the patient experience with their care or health services, and PROMs, pertaining to patient’s health, quality of life, or functional status associated with illness or treatment.

A fundamental consideration in the development of an instrument assessing PRM is the definition of the concept to be measured with domains that must be explored. As mentioned by the Cochrane Handbook for Systematic Reviews of Interventions: “Concepts may relate to an individual item or to a subset of items that refer to the same concept, often referred to as domains. For example, an item assessing difficulty walking up stairs would be a concept related to physical functioning and might be labelled walking up stairs or as part of physical function” [8]. So a domain could be seen as a concept component or a sub-part of a concept. A dimension is an item or a subset of items that explores a domain. For example, health-related quality of life is a multidimensional concept. This concept is traditionally composed of several domains including the social domain, which could be explored by different dimensions like “activities” or “family” dimensions. Currently, concepts and domains that should be explored to capture chronic patient experience remains to be identified. A first step in adequately conceptualizing chronic patient experience is to identify all essential core concepts and components that are currently measured with existing PRMs for chronic patients. This critical step could help researchers that would wish to evaluate full or partly patient experience of chronic disease. It can be valuable in different manners: draw a picture of concepts and domains related to patient experience in chronic illness, identification of lack of evaluation of some domains and development of new questionnaires filling this lack, and avoid development of new questionnaires if questionnaires assessing the same concepts already exist. The purpose of this review was to collect and explore patient reported measures (PROMs or PREMs) instruments, validated for a specific chronic disease or for any chronic patients, to produce an inventory of concepts explored inside these questionnaires, and to identify all categories of dimensions that allow capturing global chronic patient experience.

## Methods

Following recommendations published by Moher and al. [9], a systematic review of PRM instruments validated for chronic patients was conducted from November 2016 to December 2016, on articles published until November 2016. No starting date has been specified to avoid missing some primary validation studies of questionnaires. It comprised three stages consisting of: 1) Search strategy: identification of articles by specifying inclusion and exclusion criteria, keywords and search strings in the databases, 2) Selection: article pre-selection by reading titles, followed by a selection by reading abstracts and full-text, 3) Extraction: extraction of data from articles, filling in a reading grid and providing a synthesis.

### Stage 1: Search strategy

Relevant articles were searched from three databases likely to deal with PRM (Medline, the Cochrane library, and Psycinfo). Because the use of searchable technical terms for indexing international literature in databases is not always up-to-date, the search strategy was composed of free text terms, synonyms, and MeSH terms. Before building the search, some well-known concepts in PRM were defined by consensus between the authors based on the literature. As PROMs was not a Mesh term in 2016, other MeSH terms and free text terms dealing with PROMs concepts (“*Patient Outcome Assessment*”, “*Quality of life” (QOL),* “*Health Related Quality Of Life” (HRQOL)*, “*Attitude to Health”, “Illness Behavior”, “Activities of Daily Living*”…) were included into the search equation [see Additional file 1]. PREMs’ terms, often approached by the “*patient centered care*” expression [10] and used to capture the overall patient experience of health care or health service were also added to the search equation, such as the “*patients’ satisfaction*” MeSH term. This last term appeared to be a distinct but important PRM concept with a predominantly affective judgment formed by the patient [11].

In brief, the search string subsumes five blocks: the first one related to chronic patients, the second one related to the different kinds of PRMs concepts identified, the third related to measurement instruments, the fourth related to the primary validation study and the last one indicating exclusion criteria. The primary inclusion and exclusion criteria are shown in Table 1. Because the focus was on the primary validation of questionnaires, studies that reported translation and transcultural validation, revised scale validation, scale revalidation or only scale use were excluded. The focus was on adults with chronic diseases, defined as diseases of long duration and generally slow progression according to the World Health Organization [12]). Questionnaires for the patient’s family (including proxy instruments) were excluded as well as instruments for children, for patients with acute disease, or patients with psychiatric disease.

**Table 1:** Article eligibility criteria

Inclusion criteria:	Exclusion criteria:
- Full text original article - Questionnaire for patients with chronic disease - Questionnaire to measure patient reported outcomes (PROMs), patient reported experiences (PREMs) or patient satisfaction - Report of a scale construction, evaluation and validation of psychometric properties (primary study) - Published in English or French - Published until November 2016	- Questionnaires to evaluate a patient with acute or psychiatric disease - Questionnaires to evaluate children or a patients family - Short or revised form of a scale (including additional modules) - Transcultural adaptation or translation validation studies - Scale revalidation on another sample or deepening of scale psychometric properties - Articles exclusively related to content and face validation - Studies using a scale without performing any validation - Comparisons of scale psychometric properties - Scale systematic review articles - Instruments with a predominantly diagnostic, screening, prognostic or utility purpose

### Stage 2: Article selection

To select articles, a first author reviewed the titles of all records retrieved from the initial search. Then a second and a third author performed an independent review of the same articles by sharing the full list of titles. There were two kinds of disagreements: those related to inclusion or exclusion of articles and those related to the reason for exclusion. The two reviewers in question resolved the disagreement by mutual agreement after referring to the abstract. Once articles were selected by title, the same procedure was used to score the available abstracts and full-text articles, using the same article selection and disagreement resolution process.

### Stage 3: Data extraction

Data from selected articles were extracted and uploaded to a reading grid to classify the different questionnaires and their dimensions. Articles original objective (targeted concept), targeted population, questionnaires’ dimensions, language, name and year of publication were collected. To begin, all selected questionnaires were classified into different groups according to the targeted concept mentioned initially by the authors of each validation article. Once the questionnaires were classified according to their targeted concepts, their dimensions were sorted into different categories. The names of the dimensions were also based on report of the original authors, whereas dimensions classification (categories of dimensions) has been created by authors of the systematic review according to the description of these dimensions and the content of their items when they were detailed. Once again the two reviewers in question resolved the disagreement by mutual agreement if necessary.

Given the broad family of measures in PROMs, different categories of dimensions were expected based on HRQOL, functional status, and patient experience of treatment. HRQOL is mainly defined as “the assessment of the impact of disease and treatment across the physical, psychological, social, and somatic domains of functioning and well-being” [13]. Given this, *“physical” (physical symptoms, pain or discomfort), “psychological” (feelings and self-esteem), and “social*” *(social environment, activities, relationships and support)* categories of dimensions were expected. A “*functional*” dimension category *(including mobility, dependence, work capacity and impact on activities of daily living)* was also added for questionnaires exploring *functional status* which is defined as *“*an individual’s effective performance of or ability to perform those roles, tasks, or activities that are valued (e.g. going to work, playing sports, or maintaining the house)” [1]. Two categories of dimensions called *“effects of treatment”* (benefic or side effects) and *“expectations and satisfaction with treatment”* dimensions were expected for questionnaires focusing on patient experience of treatment. Finally, a last category of dimensions more related to PREMs and called *“experience of healthcare” (including involvement in decisions and respect for preferences, accessibility and continuity of care, physicians communication, support and trust)* was created, bringing a total of seven initial potential categories of dimensions. New categories of dimensions could be added if they have not been considered ahead of the reading grid development. At the end, categories which were similar enough were merged. In theory, a dimension explores a particular domain, but in practice this was not necessarily the case. Several questionnaires were composed of composite dimensions dealing with several domains. As a consequence, we have been forced to classify certain dimensions into several categories of dimensions if appropriate (for example, a dimension called “*physical symptoms and everyday living*” could be classified both in “*physical*” and in “*functional*” categories of dimensions).

### Statistical analysis

To evaluate whether the reviewers agreed with each other, Kappa coefficients were computed. This allowed to estimate consistency related to inclusion and exclusion of articles at each selection step [14]. Descriptive statistical analyses (medians, quartiles, minimum, maximum and frequencies) for the main variables of the extraction reading grid were performed. The analysis was performed using software R 2.12.1.

## Results

### Selection and description of articles

**Fig. 1:**
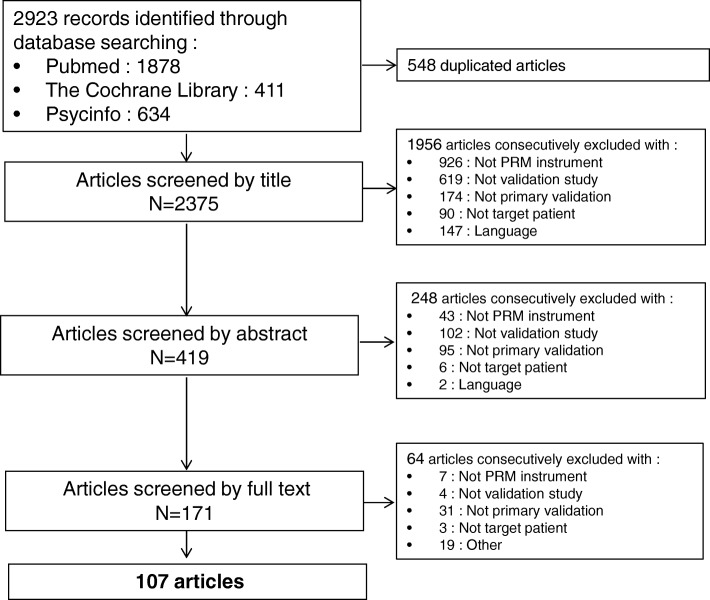
Preferred Reporting Items for Systematic Reviews and Meta-Analyses (PRISMA) flow diagram of the selection process. **Not PRM instrument:** Articles which don’t deal with a patient reported measure (PRM) (including studies with proxy instrument or questionnaire to assess family experience or healthy people point of view on illness); **Not validation study:** Studies using PRM without performing any validation (including comparisons of scale psychometric properties or scale systematic review articles); **Not primary validation:** Short or revised form of a PRM, transcultural adaptation or translation validation studies, scale revalidation on another sample or deepening of scale psychometric properties, and articles exclusively related to content and face validation; **Not target patient:** questionnaires to evaluate children or patient with acute or psychiatric disease; **Language:** Articles not published in English or French; **Other:** Unavailable articles or questionnaire’s dimensions not mentioned in full text

Figure 1 lists the process of literature identification, screening for eligibility, and selection of studies during the literature search presented in a Preferred Reporting Items for Systematic Reviews and Meta-Analyses (PRISMA) flow diagram [9]. The search string identified a total of 2923 potentially relevant articles that met the search criteria in the three bibliographic databases. After the removal of duplicates (*n* = 548) the title content of 2375 studies published from 1976 to 2016 was reviewed for eligibility. After the pre-selection step including assessment of abstracts (*n* = 419) and full-text articles (*n* = 171), 107 studies that investigated the measurement properties of instruments related to patient experience in chronic illness were selected. Kappa coefficients between authors during the different selection process ranged from 0.79 to 0.88.

The earliest retained study of the review was published in 1980 and the latest in 2016. The median year of publication was 2005, with a first quartile year of 2000 and a third quartile year of 2010.

**Fig. 2:**
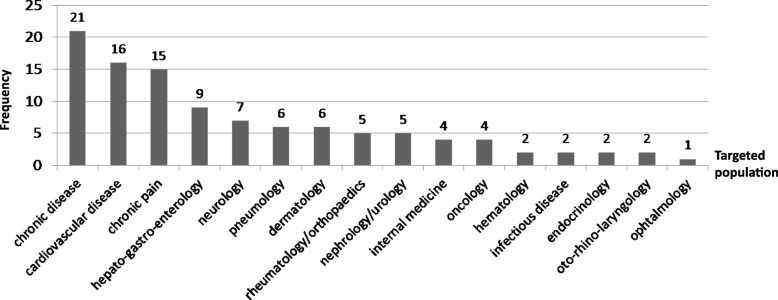
Targeted population of the 107 selected questionnaires. **Chronic disease:** questionnaires intended for patients with various chronic diseases**; Cardiovascular disease:** chronic-venous-insufficiency, leg wound (with either venous or arterial ulcers), chronic-heart-failure, coronary-heart-disease, hypertension, atrial-fibrillation; **Chronic pain**: according to the questionnaires: patients with pain at different sites (headache or facial pain, musculoskeletal-pain, back or cervical pain, extremity pain, thoracic or abdominal pain, fibromyalgia…), specific or generalized, current continuous or intermittent; **Hepato-gastro-enterology**: gastro-esophageal reflux, irritable bowel syndrome, chronic-diverticular-disease, constipation, chronic-liver-disease, hepatic-encephalopathy; **Pneumology**: chronic respiratory disease, chronic obstructive pulmonary disease, asthma, chronic-cough; **Neurology**: vertigo, epilepsy, stroke, multiple-sclerosis, amyotrophic lateral sclerosis, progressive-neuromuscular-disease, spinal-cord-injury, Parkinson’s disease; **Dermatology**: chronic skin disorders, psoriasis, eczema, urticaria, ichthyoses, seborrheic dermatitis, lichen planus, chronic oral mucosal condition, recurrent aphthous stomatitis, vesiculobullous conditions (mucous membrane pemphigoid and pemphigus vulgaris), granulomatosis, recurrent genital herpes; **Rheumatology/Orthopaedics**: chronic-rheumatic-diseases, rheumatoid-arthritis, shoulder-problem, chronic-whiplash-associated-disorders; **Nephrology/ Urology**: chronic kidney diseases, hemodialysis, chronic-prostatis, intermittent-self-catheterization; **Internal medicine**: lupus, sarcoidosis, chronic-fatigue, syncope; **Oncology**: various kinds of cancers, chronic radiation patients; **Hematology**: sickle-cell-disease, chronic graft-versus-host disease; **Infectious diseases**: human immunodeficiency virus (HIV); **Endocrinology**: diabetes, thyroïd-eye-disease; **Otorhinolaryngology**: chronic otitis media, chronic-nasal-dysfunction; **Ophtalmology**: glaucoma

The target population of the final selected questionnaires was patients with various chronic illnesses in 19.6% of the cases (*n* = 21). These 21 instruments were developed to be used across a broad spectrum of diseases rather than validated for a specific disease. Some other questionnaires (14.0%, *n* = 15) were validated on individuals with chronic pain where the pain could be related or not to a specific location. Lastly, other selected instruments were designed for specific patients in various medical specialties (the most frequent specialty was cardiology, *n* = 16) (Fig. 2).

### Targeted concepts of the 107 selected questionnaires

The 107 selected questionnaires were classified into five groups. The first group was composed of 29 questionnaires (27.1%) dealing with *HRQOL or QOL* concepts. The second group (19 questionnaires, 17.8%) focused on more specific PROMs concepts that were *symptoms* and *functional status*. The 20 questionnaires (18.7%) of the third group were developed to analyze concepts related to patient’s *feelings and attitudes about illness.* The fourth group (19 questionnaires, 17.8%) evaluated concepts related to patient’s *attitudes about treatments or healthcare.* And the last group (20 questionnaires, 18.7%) gathered questionnaires that explored patients’ *experience of treatments or healthcare*.

### Dimensions explored according to questionnaire targeted concepts

#### Questionnaires assessing HRQOL or QOL (Fig. 3a)

The classification of each dimension of the 29 selected questionnaires assessing *HRQOL* or *QOL* is shown in Additional file 2. Authors often used the briefer and more common abbreviation *QOL* as a synonym for *HRQOL* which was the most frequently explored concept in the selected questionnaires (*n* = 25, 23.4%). Dimensions observed in *HRQOL* questionnaires were mainly “*physical*” dimensions (*physical symptoms, pain*…), “*psychological*” dimensions (*depression, anxiety, mood, emotions*…), “*social*” dimensions (*social activities, work, leisure, relationship, family, friends, community, social support, environment, isolation, financial status*…), and “*functional*” dimensions (*impact of symptoms, disability, patient’s autonomy and limitations to realize activities of daily living: food, sleep, memory, concentration, sexual disorders, emotional and social functioning*…) [15–28]. Most questionnaires were structured using these four dimension categories, even if this categorization was not always explicitly defined [29–32]. If some authors agreed that a disease affects patients in a multidimensional manner, others argued that all dimensions cannot be covered exhaustively by a short and useful instrument [33]. In some instances, some aspects of health related quality of life were considered particularly important [23, 34, 35] and all dimensions were not explored. Some authors tried to explore all *HRQOL* aspects in a unique global *HRQOL* dimension [33, 36, 37] with the goal to assess in itself broader concepts like “*overall quality of life*” [34] or “*general health*” [20]. This kind of dimensions has been gathered in a new category called “*global dimensions*”. *Health status* was also a concept sometimes mistakenly interchanged with *HRQOL* or *QOL*. Four questionnaires presented *health status* as the main explored concept [38–41]. *Health status* instruments more likely measure deviations from a state of health, or from the absence of illness and disease [42]. However in this review, one health status instrument also contains a “*QOL*” dimension [41]. Lastly, two instruments were divided arbitrarily in two distinct dimensions, each evaluating *HRQOL* and *symptoms* [43, 44].

**Fig. 3:**
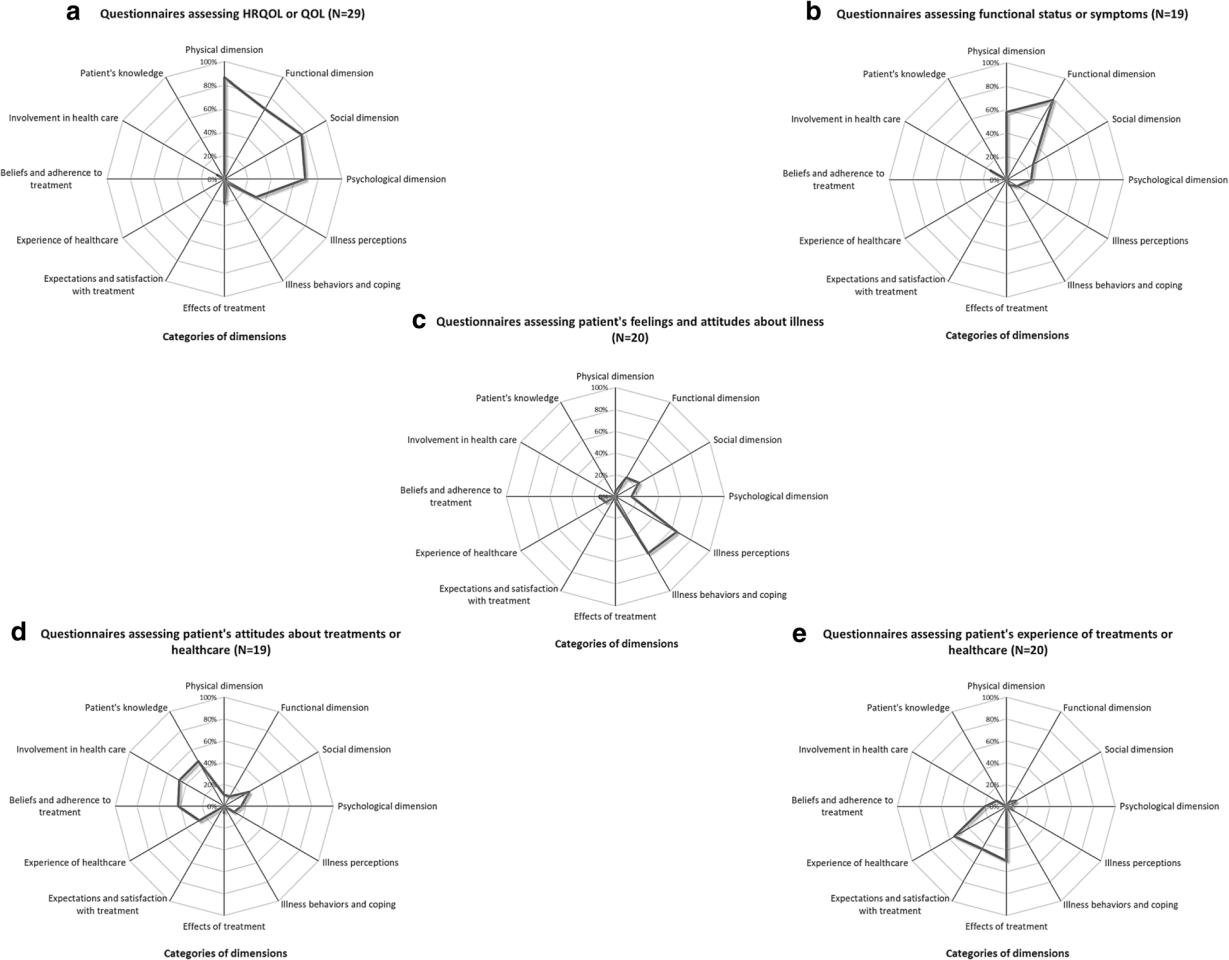
Dimensions explored according to questionnaires targeted concepts: **a** HRQOL or QOL, **b** Functional status or symptoms, **c** Feelings and attitudes about illness, **d** Attitudes about treatments or healthcare, **e** Experience of treatments or healthcare. This figure represents each dimension category and its mapping to the concept(s) to visualize which categories of dimensions were the most explored according to questionnaires targeted concepts. For example, on (**a**), 86% of the selected HRQOL or QOL instruments explored a physical dimension, 69% explored a functional dimension, 76% explored a social dimension and 69% explored a psychological dimension. HRQOL: Health-Related Quality Of Life, QOL: Quality Of Life

#### Questionnaires assessing functional status or symptoms (Fig. 3b)

Nineteen questionnaires were designed specifically to focus on *symptoms* and/or on *functional status*. The classification of each dimension of these questionnaires is shown in Additional file 3. Eight questionnaires (6.5%) were constructed to explore *symptoms* (*symptom burden, frequency, severity, duration, pain, lack of energy*…) [45–52]*.* Nine questionnaires (8,4%) explored *functional status* (*mobility, impairment, daily, household activities*…) [53–61]^*.*^

The co-evaluation of “*Functional*” and “*physical*” dimensions was frequent within these questionnaires and 2 instruments were designed to explore both *symptoms* and *functional status* simultaneously [62, 63]*.*

#### Questionnaires assessing patient’s feelings and attitudes about illness (Fig. 3c)

The classification of each dimension of the 20 questionnaires assessing patient’s *attitudes about illness* is shown in Additional file 4. These questionnaires contained predominantly two kinds of dimensions. On one hand, dimensions that were termed “*illness perceptions*” dimensions explored patients’ experience of illness [64], patients’ spirituality [65], worries or concerns about recurrence [66], health related hardiness [67], patient’s discomfort [68], patient’s burden [69], patients’ feelings of being a burden to their caregivers [70], or social impact and stigma induced by the disease [71].

On the other hand, dimensions that were termed “*illness behaviour and coping*” dimensions assessed patients’ attitude about illness [72–74], patients’ cognition [75, 76], acceptance of chronic health condition [77] and patients’ attitudes of coping [78–81]. These two categories of dimensions seemed inextricably linked and in some questionnaires both patient’s feelings about the disease and capacity to overcome this difficult life event were explored simultaneously [82]. This is exemplified in the Well-being in Wounds Inventory [83], composed of two dimensions: the ‘Wound worries’ dimension which reflects “a person’s cognitive responses to having a wound”; and the ‘Personal resources’ dimension which described “a set of positive behaviors (such as hope, optimism, and coping), which have been linked to personality traits”.

#### Questionnaires assessing patient’s attitudes about treatments or healthcare (Fig. 3d)

The classification of each dimension of the 19 questionnaires assessing patient’s *attitudes about treatments or healthcare* is shown in Additional file 5. Three kinds of questionnaires were obtained, each composed of different kinds of dimensions that formed three new categories of dimensions.

To begin, 8 questionnaires were composed of dimensions related to patient’s “*beliefs and adherence to treatment*”, assessing patient’s adherence [84, 85], patient’s beliefs about treatment [86] and factors that influence patient’s adherence to treatment [87–91].

Next, 4 questionnaires contained dimensions more related to “*patients’ knowledge”*, assessing patient’s misconceptions [92], knowledge about illness, lifestyle, treatment management or monitoring [93], skill and technique acquisition, health services navigation [94], allowed and recommended activities, entitlements, social and health care support [95].

Lastly, some dimensions were more related to patient *“involvement in healthcare*”. These dimensions dealing with individuals perception of their ability to reduce or manage symptoms and to their autonomy in self-care were mainly assessed in 7 questionnaires which focused on different concepts that were considered close enough to be merged (“patient activation” [96], *“*patients’ engagement” [97], *“*self-efficacy” [98], *“*self-management” [99], *“*self-care” [100]). Support for disease self-management (from family and friends, as well as support received from health care resources or workplace, media, public policy, neighborhood and community) [101] or potential barriers to self-management (like comorbidities, healthcare system and providers communication…) [102] were also sometimes evaluated in these instruments. Questionnaires containing these three new categories of dimensions relating to patient’s “*beliefs and adherence to treatment*”, “*patients’ knowledge*”, and patient’s “*involvement in healthcare*” were collected together since all of them seemed strongly linked, and dealt with patient’s knowledge and attitudes about treatments or healthcare.

#### Questionnaires assessing patient’s experience of treatments or healthcare (Fig. 3e)

The classification of each dimension of the 20 questionnaires related to patient’s *experience of treatments or healthcare system* is shown in Additional file 6.

##### Questionnaires about patient’s experience of treatment

12 questionnaires explored patient’s *experience of treatment*. Two categories of dimensions were found in these questionnaires.

Firstly, those which focused on patients’ “*expectations and satisfaction with treatment*” [103–108], exploring for example patients’ preferences [109] or treatment convenience of use [105, 110, 111], and secondly, those which focused on “*effects of treatment”* taking into consideration effectiveness [103, 105, 109], side effects [105, 107, 110], time to recover from treatment [112], level of expressed needs [113], treatment burden [114], and impact of treatment on health related quality of life [107] or on psychological well-being [111]. These two kinds of dimensions were often used conjointly in these instruments.

##### Questionnaires about patient’s experience of health care

Eight instruments contained dimensions that focused on patient’s *experience of health care*. These dimensions often focused on treatment staff communication [115, 116] (with one questionnaire specifically designed to explore patients’ communication preferences [117]) and also explored interaction with the physician [118], confidence and trust, and patients’ opinion about a physicians competency [115, 116]. Other dimensions more related to the healthcare system organization focused on delivered information, plan of care, link to community resources, care transitions [119], treatment planning [120], care coordination [121] and home care [122].

### Questionnaires targeted concepts into the twelve final categories of dimensions (Fig. 4)

Nearly half of the selected scales were designed to explore patients’ *QOL, HRQOL, health status, functional status or symptoms*. As a consequence, the most frequently explored dimensions in selected questionnaires were “*functional*”, *“physical”, “psychological”* and “*social”* dimensions. Patients’ *“illness perceptions”* was also a category of dimensions often explored and mainly assessed in two kinds of instruments: naturally in questionnaires specifically designed to explore patient’s *feelings and attitude about illness* in which “*illness behaviors and coping”* dimensions were jointly explored and unexpectedly in *HRQOL* instruments. Similarly, *“effects of treatment”* dimensions were logically present in specific questionnaires exploring patient’s *experience of treatments or healthcare* with “*expectations and satisfaction with treatment*” and *“experience of healthcare”* dimensions but also in *HRQOL* instruments. To finish, *“beliefs and adherence to treatment”, “patients’ knowledge”* and “*involvement in health care*” categories of dimensions where mainly explored in specific questionnaires focusing on patients’ *attitudes about treatments or healthcare.*

**Fig. 4:**
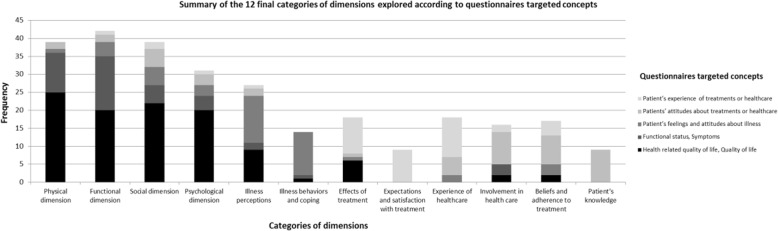
Questionnaires targeted concepts occurrence into the twelve final categories of dimensions Among the questionnaires covering Physical dimension, 25 were targeted HRQoL or QoL, 11 aimed to assess Functional status and Symptoms, 1 was targeted Patient’s feeling and attitudes about illness and 2 were targeted Patient’s feeling and attitudes about treatments or healthcare. HRQOL: Health-Related Quality Of Life, QOL: Quality Of Life

## Discussion

One hundred and seven primary PRM validation studies from 1980 to 2016 were selected. All selected instruments exploring patient experience of chronic disease or healthcare were gathered into five clusters: 1) Questionnaires exploring patients’ *HRQOL or QOL*; 2) Questionnaires focusing on *symptoms* and *functional status*; 3) Questionnaires assessing patient’s *feelings and attitudes about illness*; 4) Questionnaires assessing patients’ *attitudes about treatments or healthcare*; 5) Questionnaires related to patient’s *experience of treatments or healthcare*. After the classification of all dimensions, 12 categories were retained: the seven pre-defined categories (*“physical”, “psychological”, “social”, “functional”, “effects of treatment”, “expectations and satisfaction with treatment”,* and *“experience of healthcare”* dimensions*)* to which were added five new categories of dimensions (*“illness perceptions”,* “*illness behaviors and coping*”, *“beliefs and adherence to treatment”,* “*patients’ knowledge”,* and “*involvement in health care*” dimensions). To summarize, the twelve final categories of dimensions could be classified into three groups: the first one related to patient reported impact of illness on his/her life (with “*functional*”, *“physical”, “psychological”*, “*social”* dimensions); the second one more related to patient evaluation and satisfaction with treatment and healthcare (including “*Experience of healthcare*”, “*Effects of treatment*” and “*expectations and satisfaction with treatment”* dimensions); and a third one more related to patient’s adaptation to illness and treatment (with “*illness perceptions*”, “*illness behaviors and coping*”, “*beliefs and adherence to treatment*”, “*involvement in health care” and “patients’ knowledge”* dimensions). This representation seems to complete the Shale patient experience concept [123], outlining three main domains including: the illness experience (symptoms and illness consequences), customer service experience, and the lived experience of the illness (coping/dealing with the condition).

Inclusion of the “lived experience of the illness” seems to be an important issue to understand chronic patient experience. It has to be noted that in chronic illness, psychosocial adjustment to the disease might produce psychopathological responses resulting in mood disorders, anxiety, depression, and posttraumatic stress symptoms but can also lead to a positive adaptation phenomenon like increased ability to cope with tragedy, perceived benefits, personal growth, improved relationships [124]. In fact, if the negative aspects of changes can be assessed with the dimensions captured by this review, no concepts or dimensions focused on positive psychological change experienced by chronic patients and could be interesting to assess.

The majority of the selected questionnaires in the review were *HRQOL* instruments but the selected articles often used the terms *HRQOL, QOL* and *health status* interchangeably. If health is one of the important QOL domains, other important domains must be taken into account, and aspects of culture, values, and spirituality add to the complexity of QOL measurement [125]. Despite the increasing interest in QOL, consensus is lacking on its measurement, which could appear too broad to always be considered appropriate for the medical field. Furthermore, the terms *QOL, HRQOL* and *health status* were also often used interchangeably in the literature [126–130], even if *QOL* and *health status* are two related, but distinct concepts [131]. Thus, the coexistence of different conceptualizations or designation of the same concept is a major problem because comparability between certain tools may come into question. An unclear conceptual match between a PRM instrument and an intended claim may result in problems with analysis and interpretation of study data [132].

It has to be noted that some questionnaires mixed subjective and more factual dimensions. For example, “*Effects of treatment” (*including treatment outcomes and side effects*)* and “*expectations and satisfaction with treatment*” dimensions were sometimes explored inside the same questionnaires. However, it is important to keep in mind that these two categories of dimensions don’t explore the same domains. So if these two kinds of dimensions are interesting to evaluate, they can’t be interpreted in the same way. Given the great variety of concepts and dimensions obtained in the review, the development of an instrument exploring all chronic patient experience domains appears inappropriate. It is therefore highly recommended for researchers to focus on specific domains they are interested in, to clearly define them before any instrument development and to not encompass them in broader concepts.

For reasons of comprehension, only articles written in English or French were retained during this review and for practical reasons only three databases were searched in this study. Whilst they were of course relevant, articles in other languages cannot be evaluated. The two most frequent languages in which relevant articles were excluded were German (61 questionnaires) and Spanish (12 questionnaires). Then, the search equation was built including the most well-known kinds of PRMs concepts in the medical literature and did not cover all PRMs concepts. These could contribute to a selection bias. Indeed, many selected questionnaires were *QOL* or *HRQOL* instruments. However, *QOL* and *HRQOL* are probably the oldest and most familiar PRM concepts studied in the literature (the PRO term only appeared in 1999 [133], while articles of the review were collected since 1980). Furthermore, a wide range of PRM was captured, used for a wide range of chronic patients and designed to explore many different concepts, even if some of them were not explicitly mentioned in the search equation (like patient activation concept for example). This diversity in captured questionnaires was rather reassuring and covered all concepts listed by the Cochrane Handbook for Systematic Reviews of Interventions mentioning that PROMs: “may include the signs and symptoms reported in diaries, the evaluation of sensations (most commonly classified as symptoms), reports of behaviors and abilities (most commonly classified as functional status), general perceptions or feelings of well-being, and other reports including satisfaction with treatment, general or health-related quality of life, and adherence to treatments. Reports may also include adverse or side effects” [1].

Within this study many choices were made and could be discussed. Indeed, some diseases or symptoms (e.g. syncope) could be classified into different medical specialties and many pathological clusters could be discussed. In the same way, if most HRQOL instruments have mainly focused on physical, psychological and social functioning, unfortunately, all these dimensions were not always clearly defined and easy to classify since many dimensions simultaneously explored several domains (like the “social and emotional” dimension [30] for example). As a consequence, many dimensions were classified inside different categories. *“Illness perceptions*” dimensions and “*psychological*” dimensions were also sometimes hard to distinguish. Indeed, “*worry*” or “*concerns*” dimensions could be considered as “*psychological”* dimensions. However, since they dealt more with patient subjective perception than with psychological health status, these dimensions were clustered into the patients’ *“illness perceptions*” category of dimensions.

PRM for psychiatric patients were not considered in this review because the assumption was made that the experience of patients with chronic psychiatric disorders might differ from other chronic patients. There has been extensive evidence published demonstrating the consistency of psychiatric patients’ reports about their inner feelings [134], psychiatric patient experience could be interesting to explore and could be the subject of future focused research.

## Conclusion

Patient experience in chronic illness needs to be conceptually defined before it can be accurately measured. This review listed many concepts and dimensions that could be used to assess some aspects of chronic patient experience. A great diversity in PRMs exists for chronic patients and none of the reviewed and selected questionnaires covered all identified categories of dimensions. Categories of dimensions that were retained concerned both patient’s personal experience and attitude about illness, treatment or healthcare. It appears that the definition of concepts used varied widely among researchers and some concepts were often confusing as they are used interchangeably. These terms, similar to the many-facetted “patient experience” expression, should be well-defined before any instrument development or PRMs interpretation. Before attempting to measure the chronic patient experience, researchers should perhaps construct instruments relying on strong definition of concepts and dimensions encompassing a patient’s personal experience and attitude to illness, treatment or healthcare and possibly consider a patient’s positive adaptation to correctly measure changes in their experience of chronic disease.

## Additional files

Additional file 1: Search-equation. Abbreviations: MeSH (Medical Subject Headings); MA (Major subject headings); SU (Subjects); TI (Title); AB (Abstract); TIAB (Title/Abstract). (PDF 297 kb)

Additional file 2: Questionnaires assessing HRQOL or QOL. (XLS 59 kb)

Additional file 3: Questionnaires assessing functional status or symptoms. (XLS 52 kb)

Additional file 4: Questionnaires assessing patient’s feelings and attitudes about illness. (XLS 53 kb)

Additional file 5: Questionnaires assessing patient’s attitudes about treatments or healthcare. (XLS 54 kb)

Additional file 6: Questionnaires assessing patient’s experience of treatments or healthcare. (XLS 35 kb)

## Abbreviations

HRQOL: Health-Related Quality Of Life
*MeSH*: Medical Subject Headings
PREM(s): Patient Reported Experience Measure(s)
PRISMA: Preferred Reporting Items for Systematic reviews and Meta-Analyses
PRM(s): Patient Reported Measure(s)
PROM(s): Patient Reported Outcome Measure(s)
QOL: Quality Of Life
