# Supplementary material for: A systematic review of dimensions evaluating patient experience in chronic illness
Source: Health Qual Life Outcomes. 2019 Jan 21;17:19. doi: 10.1186/s12955-019-1084-2 (PMC6341593; doi:10.1186/s12955-019-1084-2)
Supplement: Supplementary file 1 — Search-equation. Abbreviations: MeSH (Medical Subject Headings); MA (Major subject headings); SU (Subjects); TI (Title); AB (Abstract); TIAB (Title/Abstract). (PDF 297 kb) [file 12955_2019_1084_MOESM1_ESM.pdf]

Pubmed and the Cochrane library

("Chronic disease" [Mesh] OR "Disease Progression"[Mesh] OR "Recurrence"[Mesh] OR "Chronic illness" OR "Non communicable disease")

AND

("Patient reported" [TIAB] OR "Patient reported measure" [TIAB] OR "Patient reported measures" [TIAB] OR "Patient Satisfaction"[Mesh] OR "Patient satisfaction" [TIAB] OR "Patient Centered Care" [Mesh] OR "Patient experience" [TIAB] OR "Patient Outcome Assessment" [Mesh] OR "Patient reported outcome" [TIAB] OR "Patient reported outcomes" [TIAB] OR "Quality of Life" [Mesh] OR "Health Related Quality Of Life" [TIAB] OR "Illness Behavior" [Mesh] OR "Attitude to Health"[Mesh] OR "Sick Role"[Mesh] OR "Cost of Illness" [Mesh] OR "Activities of Daily Living"[Mesh])

AND

("Surveys and Questionnaires"[Mesh] OR "survey"[TIAB] OR "questionnaire" [TIAB] OR "Index" [TIAB] OR "Indicator" [TIAB] OR "Inventory" [TIAB] OR "Instrument" [TIAB] OR "Score" [TIAB] OR "Scale" [TIAB] OR "Test" [TIAB] OR "Measurement" [TIAB])

AND

("Development" [TIAB] OR "Construction" [TIAB] OR "valid" [TIAB] OR "validation" [TIAB] OR "validation studies as topic"[Mesh] OR "Reproducibility of Results"[Mesh] OR "psychometrics" [Mesh] OR "item selection")

NOT

("Translating" [Mesh] OR "Transcultural" [TIAB] OR "cross-cultural" [TIAB] OR "Infant"[Mesh] OR "child"[Mesh] OR "adolescent" [Mesh] OR "Pediatrics" [Mesh] OR "psychiatry"[Mesh] OR "Review Literature as Topic"[Mesh])

Psycinfo (through EBSCOhost):

Bloc 1:
((SU "Chronicity (Disorders)") OR (SU "Chronic Illness") OR (MA “Chronic disease”) OR (MA “Disease Progression”) OR (MA “Recurrence”) OR “Non communicable disease”)

Bloc 2:
((SU "Client Satisfaction") OR (SU "Client Participation") OR (SU "Client Attitudes") OR (SU "Quality of Life") OR (MA “Patient satisfaction”) OR (MA “Patient Centered Care”) OR (MA “Patient Outcome Assessment”) OR (MA “Quality of Life”) OR (TI “Health Related Quality Of Life”) OR (AB “Health Related Quality Of Life”) OR (SU "Illness Behavior") OR (MA "Attitude to Health") OR (MA “Illness Behavior”) OR (MA "Sick Role") OR (MA “Cost of Illness”) OR (MA “Activities of Daily Living”) OR (TI “Patient reported”) OR (AB “Patient reported”) OR (TI “Patient reported measure”) OR (AB “Patient reported measure”) OR (TI “Patient reported measures”) OR (AB “Patient reported measures”) OR (TI “Patient reported outcome”) OR (AB “Patient reported outcome”) OR (TI “Patient reported outcomes”) OR (AB “Patient reported outcomes”) OR (TI “Patient experience”) OR (AB “Patient experience”) OR (TI “Patient satisfaction”) OR (AB “Patient satisfaction”))

Bloc 3:
((SU "General Health Questionnaire") OR (SU "Questionnaires") OR (SU "Surveys") OR (SU "Index (Testing)") OR (SU "Inventories") OR (SU "Scoring (Testing)") OR (SU "Scaling (Testing)") OR (SU "Measurement") OR (MA “Surveys and Questionnaires“) OR (TI “Instrument”) OR (AB “Instrument”) OR (TI “Indicator”) OR (AB “Indicator”) OR (TI “Test”) OR (AB “Test”))

Bloc 4:
((SU "Test Construction") OR (SU "Test Validity") OR (SU "Item Analysis (Test)") OR (SU "Item Analysis (Statistical)") OR (SU "Test Items") OR (SU "Test Reliability") OR (SU "Testing") OR (SU "Test Performance") OR (SU "Test Bias") OR (SU "Rating") OR (SU "Psychometrics") OR (MA "validation studies as topic") OR (MA "Reproducibility of Results ") OR (MA "psychometrics") OR “item selection” OR (TI “Development”) OR (AB “Development”))

Bloc 5:
((SU "Literature Review") OR (SU "Meta Analysis") OR (SU "Psychiatry")OR (SU "Pediatrics") OR (SU "Chronically Ill Children") OR (MA “Infant “) OR (MA “child “) OR (MA “adolescent “) OR (MA “Pediatrics“) OR (MA “psychiatry“) OR (MA “Review Literature as Topic “) OR (MA “Translating “) OR (TI “Transcultural”) OR (AB “Transcultural”) OR (TI “cross-cultural”) OR (AB “cross-cultural”))

(#1 and #2 and #3 and #4) NOT #5
